# Tbet promotes CXCR6 expression in immature natural killer cells and natural killer cell egress from the bone marrow

**DOI:** 10.1111/imm.13204

**Published:** 2020-06-08

**Authors:** Antonia O. Cuff, Thibaut Perchet, Simone Dertschnig, Rachel Golub, Victoria Male

**Affiliations:** ^1^ Department of Metabolism, Digestion and Reproduction Imperial College London London UK; ^2^ Unité Lymphopoïèse, Institut Pasteur, INSERM U1223 Université Paris Diderot Paris France; ^3^ UCL Institute of Immunity and Transplantation University College London London UK

**Keywords:** bone marrow, CXCR6, migration, natural killer cells, Tbet

## Abstract

Tbet‐deficient mice have reduced natural killer (NK) cells in blood and spleen, but increased NK cells in bone marrow and lymph nodes, a phenotype that is thought to be the result of defective migration. Here, we revisit the role of Tbet in NK cell bone marrow egress. We definitively show that the accumulation of NK cells in the bone marrow of Tbet‐deficient *Tbx21* ^−/−^ animals occurs because of a migration defect and identify a module of genes, co‐ordinated by Tbet, which affects the localization of NK cells in the bone marrow. *Cxcr6* is approximately 125‐fold underexpressed in *Tbx21* ^−/−^, compared with wild‐type, immature NK cells. Immature NK cells accumulate in the bone marrow of CXCR6‐deficient mice, and CXCR6‐deficient progenitors are less able to reconstitute the peripheral NK cell compartment than their wild‐type counterparts. However, the CXCR6 phenotype is largely confined to immature NK cells, whereas the Tbet phenotype is present in both immature and mature NK cells, suggesting that genes identified as being more differentially expressed in mature NK cells, such as *S1pr5*, *Cx3cr1*, *Sell* and *Cd69*, may be the major drivers of the phenotype.

## Graphical abstract

Here, we definitively show that the accumulation of NK cells in the bone marrow of Tbet‐deficient *Tbx21* ^−/−^ animals occurs because of a migration defect and identify a module of genes, co‐ordinated by Tbet, which affects the localization of NK cells in the bone marrow.

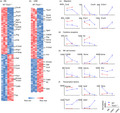

**Abbreviations**
APC: allophycocyanin
*α*LP: *α* lymphoid progenitor
CLP: common lymphoid progenitor
DAPI: 4',6‐diamidino‐2‐phenylindole
FITC: fluorescein isothiocyanate
ILC1: type 1 innate lymphoid cell
iNK: immature NK cell
LSK: lineage‐negative, Sca1^+^ ckit^+^ bone marrow progenitor
mNK: mature NK cell
NK: natural killer
PBS: phosphate‐buffered saline
PE: phycoerythrin
PerCP: peridinin chlorophyll protein
preNKP: pre‐NK progenitor
rNKP: refined NK progenitor

## Introduction

Tbet was originally described as the key transcription factor directing T helper type 1 lineage commitment.^1^ More recently, it has become clear that Tbet also drives differentiation and memory cell generation in a number of lymphocyte lineages^2^ as well as being required for the development and survival of type 1 innate lymphoid cells (ILC1).^3^ There is still debate about the extent to which ILC1 form a separate lineage from natural killer (NK) cells,^4^, ^5^, ^6^, ^7^, ^8^ with one key factor that distinguishes NK cells from ILC1 being the greater extent to which ILC1 depend upon Tbet for their development.^9^, ^10^ Nevertheless, in two strains of Tbet‐deficient mice – *Tbx21* ^−/−^ ^11^ and Duane, in which a point mutation in *Tbx21* leads to underexpression of the protein by a factor of approximately four‐fold^12^ – NK cell number, maturation status and function are abnormal.

Both strains of Tbet‐deficient mice have reduced NK cells in blood and spleen, but increased NK cells in the bone marrow and lymph nodes.^11^, ^12^ These observations led to the suggestion that Tbet is required for NK cells to leave the bone marrow and lymph nodes and enter the blood.^12^ Duane NK cells express lower levels of *S1pr5* mRNA than their wild‐type counterparts, and *S1pr5* knockout mice phenocopy Duane mice, suggesting that Tbet mediates bone marrow and lymph node egress by up‐regulating S1PR5 expression.^12^ However, a defect in NK cell migration in the absence of Tbet has not yet been formally shown, nor have potential mediators of egress other than S1PR5 been identified.

Here, we report that in the absence of Tbet, NK cells display a defect in their ability to leave the bone marrow, and in their ability to differentiate to the final stage of NK cell development. We identify a module of genes whose expression is regulated by Tbet and find that, in the absence of Tbet, a subset of CXCR6‐expressing bone marrow NK cells is lost. We also observe an accumulation of immature NK cells in the bone marrow in the absence of CXCR6, although this is smaller than that observed in the absence of Tbet, and a reduced ability of CXCR6‐deficient bone marrow to reconstitute peripheral NK cell compartments, suggesting that CXCR6 may also have a minor role in NK cell trafficking.

## Materials and methods

#### Mice

B6.129S6‐Tbx21^<tm1Glm>^/J (RRID IMSR_JAX:004648; ‘*Tbx21* ^−/−^‘) mice were purchased from the Jackson Laboratory (Bar Harbor, ME). Tbx21^−/−^ mice were crossed onto C57BL/6J mice, bred at the Royal Free Hospital, and the resultant heterozygotes were crossed to produce *Tbx21* ^−/−^ homozygote knockouts and *Tbx21* ^+/+^ homozygote wild‐type littermate controls. The absence of Tbet in *Tbx21* ^−/−^ mice at all stages of NK cell development was confirmed by flow cytometry (see Supplementary material, Figure S1). Mice were killed between 6 and 12 weeks of age with direct cervical dislocation. Death was confirmed by cessation of circulation. The hind leg bones (tibiae and femurs) and spleen were dissected to isolate leucocytes. Animal husbandry and experimental procedures were performed according to UK Home Office regulations and institute guidelines under project licence 70/8530.

B6.129P2‐*Cxcr6* ^tm1Litt^/J (RRID MGI:3616633; ‘*Cxcr6* ^gfp/+’^ and ‘*Cxcr6* ^gfp/gfp^’)^13^ and Rag2^−/−^ *γ*c^−/−^ mice were bred in the animal facilities at the Pasteur Institute, Paris. Mice were cared for in accordance with Pasteur Institute guidelines in compliance with European animal welfare regulations, all animal studies were approved by the Pasteur Institute Safety Committee according to the ethical charter approved by the French Agriculture Ministry and the European Parliament Directive 201B0/63/EU. Work was carried out under project 02080.02 issued 5 May 2016. Mice were killed between 8 and 12 weeks of age with carbon dioxide pump. Death was confirmed by cessation of circulation. The hind leg bones (tibiae and femurs) and spleen were dissected to isolate leucocytes.

#### Bone marrow transplantation

For competitive reconstitution experiments with CXCR6‐deficient progenitor cells, recipient Rag2^−/−^ *γ*c^−/−^ mice were non‐lethally irradiated (400 rad) at least 4 hr before cell injection. Recipients were reconstituted with a mixture of 5000 sorted CD45.1 wild‐type LSK cells (Lineage‐negative, Sca1^+^ ckit^+^ bone marrow progenitor cells) and 5000 sorted CD45.2 wild‐type, *Cxcr6* ^gfp/+^ or *Cxcr6* ^gfp/gfp^ LSK cells. Mice were analysed 8 weeks after transplant.

#### Sinusoidal cell labelling

Mice received an intravenous injection of 1 µg anti‐CD45.2 phycoerythrin (PE)‐eFluor 610‐conjugated monoclonal antibody (eBioscience, San Diego, CA) in phosphate‐buffered saline (PBS; Life Technologies, Thermo Fisher Scientific, Waltham, MA). Mice were killed after 2 minutes.

#### Cell isolation

Bone marrow was flushed from dissected bones with supplemented RPMI‐1640 medium (Life Technologies). Tissue clumps were pelleted at 500 *g*, 4° for 5 min, then mechanically disaggregated and filtered through a 70‐µm cell strainer. RPMI‐1640 medium was supplemented with 10% fetal calf serum, non‐essential amino acids (100 µm each), 1 mm sodium pyruvate, 50 U/ml penicillin–streptomycin, 25 mm HEPES buffer and 50 µm 2‐mercaptoethanol (all from Life Technologies).

Spleens were passed through a 40‐µm cell strainer. Red blood cells were lysed by incubation in ACK lysing buffer for 5 min at room temperature. Intrahepatic lymphocytes were isolated by collecting finely minced liver tissue in RPMI 1640 medium and passing the cells through a 70 μm cell strainer. The suspension was spun down (500 *g*, 4°, 10 min) and the pellet was resuspended in RPMI‐1640 medium. The cell suspension was layered over 24% Optiprep (Sigma‐Aldrich, St Louis, MO) and centrifuged without braking (700 *g*, room temperature, 20 min). The interface layer was taken and washed in Hanks’ balanced salt solution without Ca^2+^ Mg^2+^ (Lonza, distributed by VWR, Lutterworth, UK) supplemented with 0·25% bovine serum albumin (Sigma‐Aldrich, Hammerhill, UK) and 0·001% DNase I (Roche, distributed by Sigma‐Aldrich).

For cell sorting on a FACSAria (BD Biosciences, Oxford, UK), total bone marrow cells were incubated in ACK lysing buffer (Life Technologies) for 5 min at room temperature to lyse red blood cells and to enrich for leucocytes before cell sorting. The leucocytes were further enriched for NK cells by immunomagnetic depletion of lineage‐associated cells (CD3, CD8*α*, CD19, Gr‐1) using the relevant fluorescein isothiocyanate (FITC) ‐conjugated antibodies and anti‐FITC MicroBeads (Miltenyi, Woking, UK). The sorting buffer consisted of PBS, 0·5% bovine serum albumin (Sigma Aldrich) and 2 mm EDTA (Sigma Aldrich).

#### Flow cytometry

The following anti‐mouse antibodies were used: anti‐CCR2‐PE‐Cyanine7 (clone SA203G11, Biolegend, London, UK), anti‐CD3‐FITC (17A2, Biolegend), anti‐CD3‐biotin (clone 145‐2C11, Sony Biotechnology, Surrey, UK), anti‐CD4‐BV786 (clone RM4‐5, BD Horizon), anti‐CD8*α*‐FITC (clone 53‐6.7, Biolegend), anti‐CD8*α*‐biotin (clone 53‐6, Sony), anti‐CD11b‐allophycocyanin (APC) (clone M1/70, Biolegend), anti‐CD11b‐PE (clone M1/70, Biolegend), anti‐CD11b‐AlexaFluor700 (clone M1/70, BD Pharmingen), anti‐CD19‐FITC (clone 6D5, Biolegend), anti‐CD19‐Peridinin chlorophyll protein (PerCP)‐Cyanine5.5 (clone 6D5, Biolegend), anti‐CD19‐biotin (clone 6D5, Sony), anti‐CD27‐APC (clone LG.3A10, Biolegend), anti‐CD27‐APCeFluor780 (clone LG.7F9, eBioscience), anti‐CD27‐PE‐Dazzle594 (clone LG.3A10, Biolegend), anti‐CD27‐APC‐Cyanine7 (clone LG.7F9, eBioscience), anti‐CD45‐BV510 (clone 30‐F11, Biolegend), anti‐CD45‐APC‐Cyanine7 (clone 30‐F11, BD Pharmingen), anti‐CD45.2 PE‐eFluor610 (clone 104, eBioscience), anti‐CD45.1‐PE‐Cyanine7 (clone A20, Biolegend), anti‐CD49a‐BUV395 (clone Ha31/8, BD Bioscience), anti‐CD49b‐PerCP‐eFluor710 (clone DX5, eBioscience), anti‐CD49b‐BV510 (clone HMα2, BD Optibuild), anti‐CD62L‐PerCP‐Cyanine5.5 (clone MEL‐14, Biolegend), anti‐CD69‐PerCP‐Cyanine5.5 (clone H1.2F3, Biolegend), anti‐CD117(cKit)‐BV510 (clone 2B8, Biolegend), anti‐CD117 (ckit)‐APC‐Cyanine7 (clone ACK2, Sony), anti‐CD122 (IL‐2R*β*)‐eFluor450 (clone TM‐*β*1, eBioscience), anti‐CD122 (IL‐2R*β*)‐APC (clone TM‐*β*1, Biolegend), anti‐CD127 (IL‐7R*α*)‐PE (clone A7R34, eBioscience), anti‐CD127 (IL‐7R*α*)‐PE‐Cyanine7 (clone A7R34, Sony), anti‐CD135 (Flt3)‐PerCP‐eFluor710 (clone A2F10, eBioscience), anti‐CD135 (Flt3)‐PE (clone A2F10, Sony), anti‐CD244.2‐ PE‐Cyanine7 (clone m2B4 (B6)458.1, Biolegend), anti‐CX3CR1‐BV510 (clone SA011F11, Biolegend), anti‐CXCR6‐PE‐Cyanine7 (clone SA051D1, Biolegend), anti‐Gr‐1‐FITC (clone RB6‐8C5, Biolegend), anti‐Gr‐1‐APC (clone RB6‐8C5, Biolegend), anti‐Gr‐1‐biotin (clone RB6‐8C5, BD Pharmingen), anti‐integrin *α*_4_ *β*_7_‐BV421 (clone DATK32, BD Horizon), anti‐NK1.1‐FITC (clone PK136, Biolegend), anti‐NK1.1‐APC‐eFluor780 (clone PK136, eBioscience), anti‐NK1.1‐BV650 (clone PK136, Sony), anti‐NKp46‐V450 (clone 29A1.4, BD Horizon), anti‐Sca‐1‐AlexaFluor700 (clone D7, Biolegend), anti‐Sca‐1‐BV510 (clone D7, Sony), anti‐S1PR5‐PE (clone 1196A, Bio‐Techne, Abingdon, UK), anti‐TCR*αβ*‐biotin (clone H57 597, Sony), anti‐TCR*γδ*‐biotin (clone GL3, BD Pharmingen), anti‐TER119‐biotin (clone TER‐119, Sony) and streptavidin‐PE‐Cyanine5 (BD Pharmingen) for surface antigens; and anti‐Eomes‐PE‐Cyanine7 (clone Dan11mag, eBioscience) and anti‐Tbet‐eFluor660 (clone eBio4B10, eBioscience) for intracellular staining.

**Figure 1:**
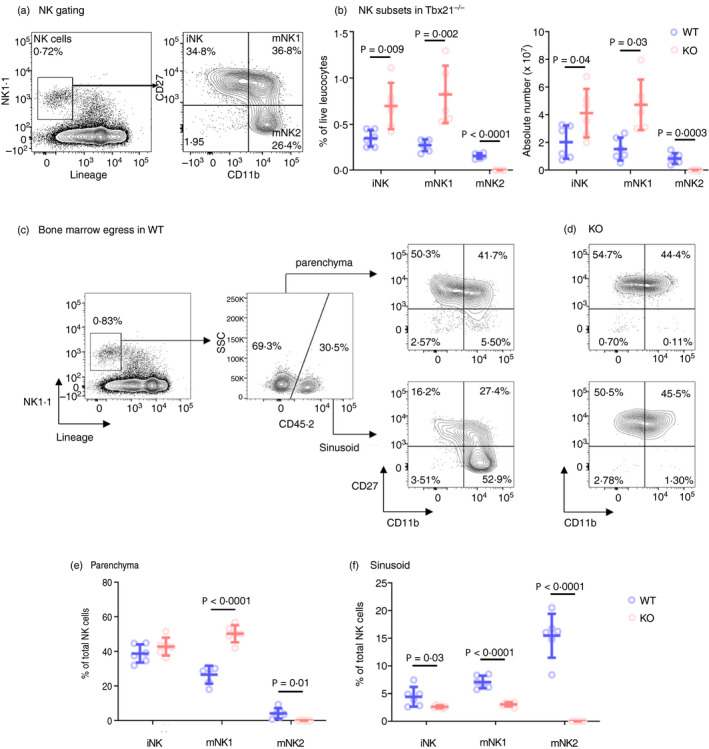
Reduced natural killer (NK) cell bone marrow egress in the absence of Tbet. (a) Flow cytometry gating strategy identifying NK cells in the bone marrow. NK cells were identified by gating on single, live, CD45^+^ cells and by leucocyte scatter, lineage negative and NK1·1^+^. Immature NK (iNK), mature NK1 (mNK1) and mNK2 cells were identified by CD11b and CD27 expression, as shown. (b) Summary graphs showing NK cell developmental stages as a percentage of live leucocytes, and absolute numbers, in *Tbx21* ^+/+^ (wild‐type; WT) compared with *Tbx21* ^−/−^ (knockout; KO) mice. (c, d) Sinusoidal NK cells were labelled *in vivo* by intravenous injection of 1 µg PE‐eFluor610‐labelled CD45.2 antibody followed by a 2‐minute incubation, as an indicator of recent egress from the bone marrow. The flow cytometry gating strategy used to identify parenchymal (CD45.2‐negative) and sinusoidal (CD45.2‐positive) cells among the lineage‐negative NK1.1^+^ gate in wild‐type (c; WT) and *Tbx21* ^−/−^ (d; KO) mice is shown. (e, f) Summary graphs showing the percentage of each subset of NK cells as a proportion of the total NK cell population in the bone marrow parenchyma (e) and sinusoid (f) of *Tbx21* ^+/+^ (WT) compared with *Tbx21* ^−/−^ (KO) mice. In the parenchyma of *Tbx21* ^−/−^ mice, mNK2 are decreased 27‐fold, relative to wild‐type controls. *n* = 6 mice per group, mean and SD are shown. Significance was determined using two‐sample, one‐tailed *t*‐tests with Holm–Sidak correction.

**Figure 2:**
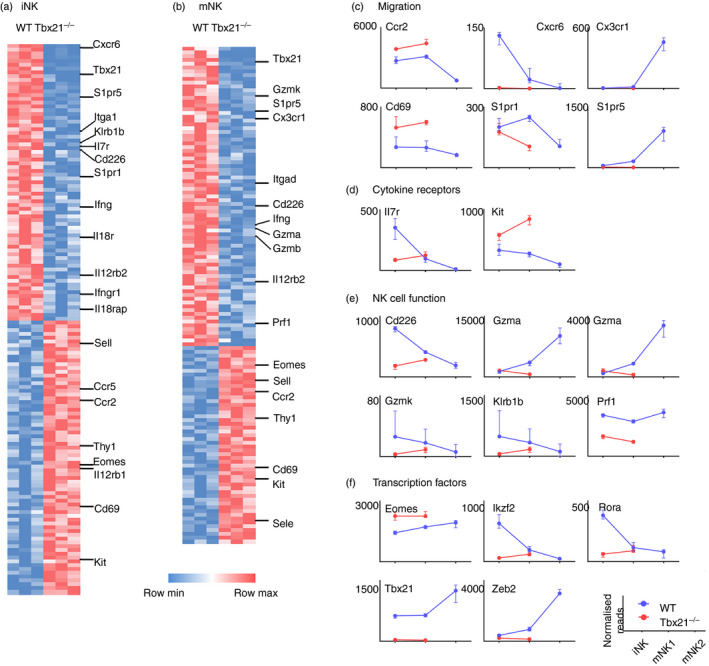
Transcriptional profile of natural killer (NK) developmental stages in the bone marrow of Tbx21^+/+^ and Tbx21^−/−^ mice. (a, b) RNASeq data from immature NK (iNK) (a) and mature NK 1 (mNK1) (b) cells sorted from *Tbx21* ^+/+^ and *Tbx21* ^−/−^ mice (*n* = 3 pairs). Differentially expressed genes were identified as those with fold change >2 and *P*_adj_ < 0·05. (c–f) Normalized expression of the transcripts of selected mediators of migration (c), cytokine receptors (d), NK cell effector proteins (e) and transcription factors (f) over the course of NK cell development are shown (medians and interquartile range; IQR).

**Figure 3:**
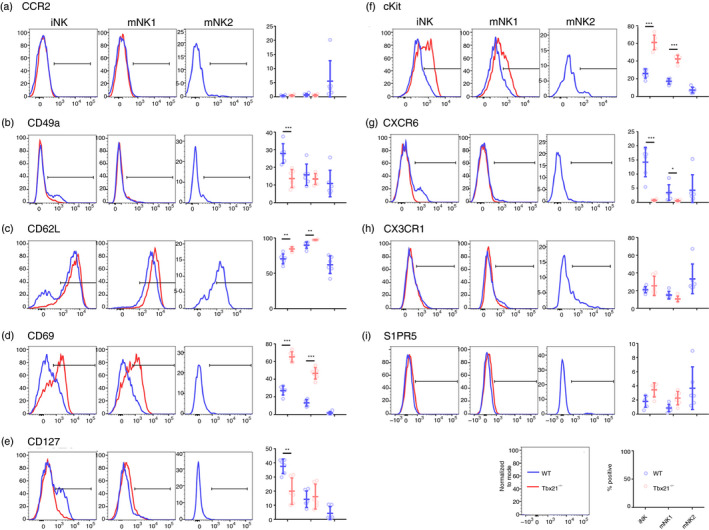
Phenotype of developing natural killer (NK) cells in the presence or absence of Tbet. Leucocytes were isolated from *Tbx21* ^+/+^ [wild‐type (WT); blue] and *Tbx21* ^−/−^ (red) bone marrow and immature NK (iNK), mature NK (mNK) 1 and 2 cell subsets were screened by flow cytometry for differentially expressed proteins. Proteins examined were selected based on differential expression of their transcripts between *Tbx21* ^+/+^ and *Tbx21* ^−/−^ in the RNASeq screen. Representative histograms and summary graphs for (a) CCR2, (b) CD49a, (c) CD62L, (d) CD69, (e) CD127, (f) cKit, (g) CXCR6, (h) CX3CR1 and (i) S1PR5 are shown. *n* = 6 mice per group, means and SD are shown. Significance was determined using two‐sample, one‐tailed *t*‐tests with Holm–Sidak correction. *P < 0·05, **P < 0·01, ***P < 0·001.

**Figure 4:**
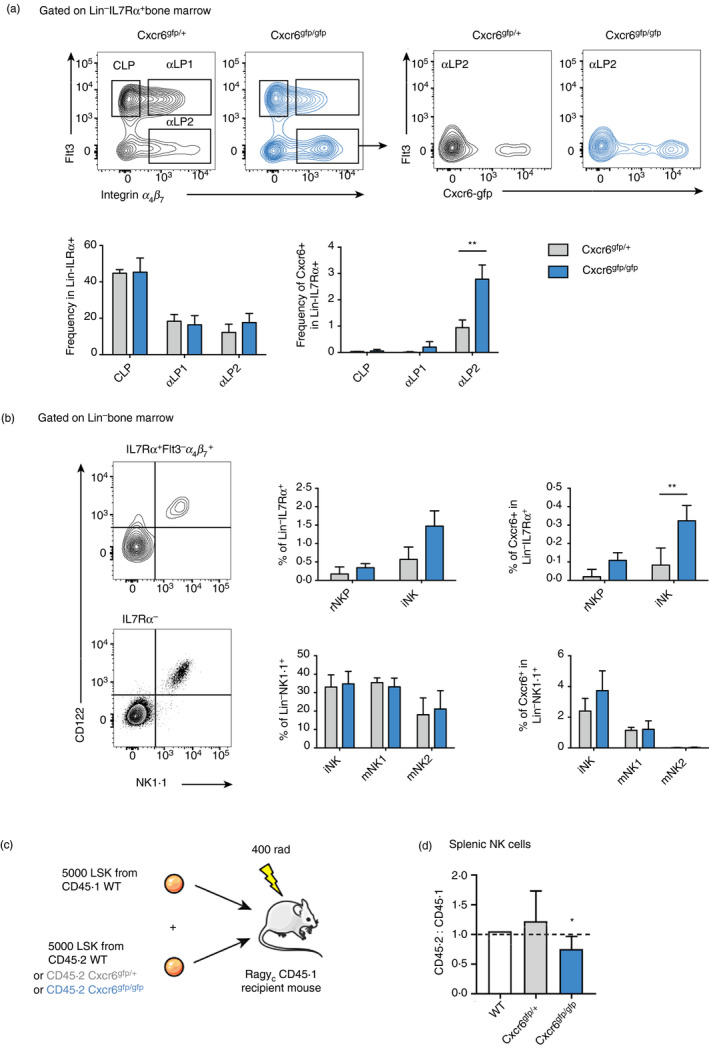
Accumulation of Cxcr6^+^ immature natural killer (iNK) cells in the bone marrow of CXCR6‐deficient mice. (a) Representative flow cytometry gating strategy used to identify common lymphoid progenitor (CLP), *α* lymphoid progenitor 1 (*α*LP1), *α*LP2 progenitors (Lin^−^ IL7R*α* ^+^) in the *Cxcr6* ^gfp/+^ and *Cxcr6* ^gfp/gfp^ bone marrow.^28^ *α*LP2 cells were identified as Flt3^−^ *α*_4_ *β*_7_^+^ cells and contour plots indicate the fraction of *Cxcr6‐gfp^+^* cells among *α*LP2 from both *Cxcr6* ^gfp/+^ and *Cxcr6* ^gfp/gfp^ mice. Frequencies of the CLP, *α*LP1 and *α*LP2 are not significantly different among total bone marrow progenitors. When progenitors are selected as *Cxcr6*‐expressing cells, accumulation of *α*LP2 fraction in the bone marrow is significant. *n* = 10 mice per group, means and SD are shown. (b) NK progenitor subsets among Lin^−^ IL7R*α* ^+^ or the Lin^−^ IL7R*α* ^−^ subsets are identified using CD122 and NK1.1 expression as shown. Refined NK progenitor (rNKP)^29^ and iNK cells are identified among the IL7R*α* ^+^ *α*LP2 fraction as CD122^+^ NK1.1^−^ and CD122^+^ NK1.1^+^ subsets, respectively. Among IL7R*α* ^−^ cells, iNK, mature NK 1 (mNK1) and mNK2 cells are identified as shown in Fig. 1. In CXCR6‐deficient mice, iNK frequency presents a tendency to increase with a significant increase for the *Cxcr6‐gfp^+^* IL7R*α* ^+^ iNK fraction. *n* = 10 mice per group, means and SD are shown. Significance was determined using two‐sample, one‐tailed *t*‐tests. *P < 0·05, **P < 0·01, ***P < 0·001. (c) Schematic representation of the experimental procedure for competitive reconstitution of congenic lymphopenic Rag/*γ*c double knockout (KO) mice. (d) The ratio of CD45.2 (wild‐type; WT), *Cxcr6* ^gfp/+^ or *Cxcr6* ^gfp/gfp^ to CD45.1 (WT cells among splenic NK cells) is shown. WT: *n* = 2 mice, *Cxcr6* ^gfp/+^ and *Cxcr6* ^gfp/gfp^: *n* = 8 mice, means and SD are shown. Significance was determined using a single‐sample, one‐tailed *t*‐test.

In Figs 1, 2, 3 and the Supplementary material (Figure S1) the lineage cocktail used to identify immature NK (iNK), mature NK1 (mNK1) and mNK2 cells consisted of anti‐CD3‐, CD8*α*‐, CD19‐ and Gr‐1‐FITC (Lin). The lineage cocktail used for the identification of lymphoid primed multipotent progenitor, common lymphoid progenitor (CLP), pre‐NK progenitor (preNKP) and refined NK progenitor (rNKP) cells (see Supplementary material, Figure S1) consisted of anti‐CD3‐, CD8*α*‐, CD19‐, Gr‐1‐ and NK1.1‐FITC. Lineage cocktail for Fig. 4 consisted of anti‐CD3‐, CD8*α*‐, CD19‐, TCR*αβ*‐, TCR*γδ*‐, Gr1‐ and TER119‐biotin, stained with streptavidin‐PE‐Cyanine5. NK1.1 was added in an another independent channel to either add it to the Lin cocktail to dump NK1.1^+^ cells for CLP, *α* lymphoid progenitor 1 and *α* lymphoid progenitor 2 cells or to stain independently of Lin‐ cells for the determination of the different stages of NK cell commitment (for rNKP, iNK). Dead cells were excluded using fixable viability dye eFluor450 (eBioscience), fixable viability dye eFluor455 ultraviolet (eBioscience), propidium iodide (Sigma Aldrich) or DAPI (4',6‐diamidino‐2‐phenylindole, Dilactate, Biolegend). Intracellular staining was carried out using human FoxP3 buffer (BD Biosciences) according to the manufacturer’s instructions.

Data were acquired on an LSRFortessa II (BD Biosciences) and analysed using flowjo (Tree Star, Ashland, OR). Identification of NK cells (as opposed to ILC1) was confirmed by the absence of Eomes^−^ CD49a^+^ cells in the gates (see Supplementary material, Figure S1). For most panels, gates were set using appropriate fluorescence minus one or internal negative controls. For *in vivo* labelling experiments, the sinusoidal gate was set using cells taken from control animals that had received PBS instead of fluorescently labelled antibody (see Supplementary material, Figure S2). Cells were sorted on a FACSAria (BD Biosciences).

#### RNA sequencing

Total RNA was extracted from sorted iNK, mNK1 and mNK2 bone marrow NK cells using an RNeasy Micro kit (Qiagen, Manchester, UK). cDNA libraries were prepared from 2 ng of total RNA (RIN 3.2‐10) using the Ovation Solo assay (NuGEN, AC Leek, The Netherlands) with 15 cycles of amplification. Libraries were assessed for correct size distribution on the Agilent 2200 TapeStation and quantified by Qubit DNA High Sensitivity assay (Thermo Fisher Scientific) before being pooled at an equimolar concentration. Sequencing was performed on the Illumina NextSeq 500, generating approximately 12 million 75‐bp single end reads per sample. Differential expression analysis was carried out using sartools ^14^ filtering at *P*_adj_ < 0·05.

#### Statistical testing

Data were assessed for normality using Shapiro–Wilk tests, in order to determine whether parametric or non‐parametric statistical tests were more appropriate. Full details of each statistical test carried out are given in the figure legends. Analysis was carried out using vassarstats (RRID SCR_010263).

## Results

### NK cells accumulate in the bone marrow of Tbx21^−/−^ mice

NK cells have previously been reported to accumulate in the bone marrow of two Tbet‐deficient strains of mice.^11^, ^12^ These previous studies either considered bulk NK cell populations^12^ or defined NK cells as immature or mature by their expression of CD11b.^11^ For a better understanding of the requirement for Tbet in NK cell development and migration, we examined the bone marrow of *Tbx21* ^−/−^ mice, further subdividing the mature NK cell population into a CD27^+^ subset of intermediate maturity, which for simplicity is sometimes called ‘mNK1’, and a more mature CD27^−^ subset called ‘mNK2’^15^, ^16^, ^17^ (Fig. 1a). This allowed us to observe an accumulation of cells in the bone marrow specifically among the iNK and mNK1 populations, with the accumulation in the mNK1 population being more pronounced (Fig. 1b). Conversely, within the mNK2 population, we observed a decrease in frequency and number of cells in *Tbx21* ^−/−^ animals, consistent with a defect at the mNK1 to mNK2 transition, which has previously been reported in this strain.^18^

### NK cells are less able to leave the bone marrow in the absence of Tbet

Previous reports attribute the bone marrow accumulation of Tbet‐deficient NK cells to a defect in migration involving S1PR5, as *S1pr5* mRNA is underexpressed in Duane NK cells, *S1pr5* ^−/−^ mice phenocopy the accumulation of NK cells in the bone marrow of Duane mice and in *S1pr5* ^−/−^ mice the accumulation is caused by a migration defect.^12^, ^19^, ^20^ However, a defect in NK cell migration from the bone marrow in the absence of Tbet has not yet been formally shown. Therefore, we examined NK cell migration from the bone marrow *in vivo*. Sinusoidal leucocyte staining has been widely used as a surrogate measure of bone marrow egress, because recently migrated cells translocate to the sinusoids as they exit the bone marrow.^12^, ^21^ The procedure involves intravenous injection of fluorescently labelled anti‐CD45.2 monoclonal antibody followed by a 2‐minute incubation to allow the circulation to carry the antibody to the sinusoids and stain leucocytes in the blood and sinusoids, while sparing parenchymal leucocytes (Fig. 1c).

As NK cells mature from CD11b^−^ iNK cells to CD11b^+^ CD27^+^ mNK1 and then CD11b^+^ CD27^−^ mNK2 cells in wild‐type mice, their frequencies decrease in the parenchyma (Fig. 1c,e) and increase in the sinusoids (Fig. 1c,f). This indicates migration out of the bone marrow occurring at all NK cell developmental stages but increasing in more mature cells. We did not observe this pattern in *Tbx21* ^−/−^ NK cells (Fig. 1d–f), whose frequency remained roughly constant between iNK and mNK1 cells in both parenchyma and sinusoid, indicating a reduced ability to migrate. The decrease in frequency of mNK2 cells in both anatomical compartments of *Tbx21* ^−/−^ bone marrow reflects the defect in the mNK1 to mNK2 transition that we observed in total bone marrow, and as described previously.^18^

### Tbet controls the expression of cell migration mediators in bone marrow NK cells

Having confirmed that the accumulation in the bone marrow of *Tbx21* ^−/−^ mice is caused by a migration defect, we sought to define the mediators involved. We therefore sorted iNK and mNK1 cells from the bone marrow of wild‐type and *Tbx21* ^−/−^ mice, and mNK2 cells (which are not present in *Tbx21* ^−/−^; Fig. 1b) from wild‐type mice and analysed them by RNAseq. Raw RNAseq data and differentially expressed gene lists are available from the National Center for Biotechnology Information Gene Expression Omnibus under accession no. GSE122874, https://www.ncbi.nlm.nih.gov/geo/query/acc.cgi?acc=GSE122874

Consistent with a previous report in the Duane mouse,^12^ we found *S1pr5* mRNA underexpressed by a factor of approximately sevenfold in both iNK (Fig. 2a,c) and mNK1 (Fig. 2b,c) cells in *Tbx21* ^−/−^ mice compared with wild‐type mice. More severely affected by the absence of Tbet was *Cxcr6*, which was underexpressed by a factor of 125‐fold in iNK cells (Fig. 2a,c). The expression of *Kit*, often considered to be a marker of iNK cells, was increased in the absence of Tbet (Fig. 2a,b,d), whereas the expression of genes associated with mature NK cell effector function, such as *Ifng*, *Gzmb* and *Prf1*, was reduced (Fig. 2a,b,e). Consistent with reports that Tbet promotes *Zeb2* ^18^ and antagonizes *Eomes* ^22^ transcription, *Zeb2* and *Eomes* expression were decreased and increased, respectively, in *Tbx21* ^−/−^ NK cells (Fig. 2a,b,f).

We next selected genes identified as differing transcriptionally between wild‐type and *Tbx21* ^−/−^ NK cells to confirm the differences in their expression at the protein level by flow cytometry (Fig. 3). The genes were selected based on a significance cut off of *P*_adj_ < 0·05 and a twofold or more differential expression between wild‐type and *Tbx21* ^−/−^ iNK and mNK1 cells. *Sell* and *Cd69* did not display twofold differences between *Tbx21* ^−/−^ and wild‐type cells, but were included in the analysis because we observed differential transcription of these genes in both iNK and mNK1 cells.

The expression of several proteins associated with cell migration differed between wild‐type and knockout NK cells. In particular, CXCR6 was expressed at a lower level by knockout iNK and mNK1 cells, compared with their wild‐type counterparts (Fig. 3g). Consistent with the RNASeq data, the most significant drop in expression was within the iNK cell compartment. CD69, which suppresses the expression of S1PR1 to retain immune cells in lymph nodes and tissues, is up‐regulated in both knockout NK subsets (Fig. 3d). CD62L (encoded by *Sell*), which is involved in leucocyte homing from the blood to tissues, also has elevated expression in knockout NK cells compared with wild‐type (Fig. 3c). We also observed a decrease in the expression of CD49a in knockout iNK cells (Fig. 3b).

*S1pr5* and *Ccr2* displayed relatively large differences at the RNA level (Fig. 2a,b), but using antibodies validated on appropriate positive control cells (see Supplementary material, Figure S2) we did not see any differences at the protein level (Fig. 3a,i). Neither did we see any significant difference in CX3CR1 expression between wild‐type and knockout cells, although the expression of this protein did increase in mNK2 cells compared with previous stages of development (Fig. 3h).

### CXCR6‐deficient NK cells accumulate in the bone marrow and are less able to reconstitute the periphery

We have previously reported that ILC progenitors require CXCR6 to leave the bone marrow,^23^ so we were intrigued by the underexpression of CXCR6 we observed in *Tbx21* ^−/−^ NK cells, which are also less able to leave the bone marrow. If CXCR6 is one of the mediators through which Tbet controls NK cell bone marrow egress, we would expect to see an accumulation of NK cells in the bone marrow of CXCR6‐deficient mice, similar to our previous observations of ILC progenitors.

To test this hypothesis, we examined NK cells in the bone marrow of *Cxcr6* ^gfp/gfp^ mice, in which both *Cxcr6* alleles are inactivated and replaced by a reporter cassette encoding GFP, or *Cxcr6* ^gfp/+^ controls. We did not observe an accumulation among the general population of CLP, *α* lymphoid progenitor 1 or *α* lymphoid progenitor 2 cells in the bone marrow of these mice, although we did observe an accumulation specifically among the *Cxcr6*‐gfp^+^ population of *α* lymphoid progenitor 2 cells (Fig. 4a). Examining NK cell progenitors, we observed an accumulation of iNK cells, although this was not significant. However, we did observe a significant accumulation specifically among the *Cxcr6*‐gfp^+^ population of iNK cells (Fig. 4b). In contrast to the Tbet knockout, we did not detect an accumulation of cells among either of the mature NK cell subpopulations in the bone marrow of CXCR6‐deficient mice, suggesting that any requirement for CXCR6 in bone marrow egress occurs relatively early in NK cell development.

Finally, we used competitive reconstitution experiments to determine the relative ability of CXCR6‐deficient cells to reconstitute the peripheral NK cell compartment (Fig. 4c). The reconstitution of splenic NK cells was reduced when mice were given progenitors that lacked both alleles of *Cxcr6*, whereas heterozygote progenitors did not differ from wild‐type cells in their reconstitution ability (Fig. 4d). Therefore, CXCR6‐deficient NK cells are also less able to seed peripheral organs, further supporting the hypothesis that CXCR6 has a role in the trafficking of NK cells from the bone marrow to the periphery.

## Discussion

In this study, we revisited the role of Tbet in mediating NK cell egress from the bone marrow during development. We observed an accumulation of NK cells in the bone marrow of *Tbx21* ^−/−^ mice, in agreement with previous reports from both *Tbx21* ^−/−^ and Duane mice.^11^, ^12^ The accumulating cells in *Tbx21* ^−/−^ mice were found within both the immature and mature NK cell populations, as defined by CD11b expression^11^ whereas those in Duane mice were described as CD27^hi^ and KLRG1^lo^.^12^ Here, we use a definition of NK cell developmental stages that uses both CD11b and CD27^15^, ^16^ to show that the accumulation occurs within the iNK and mNK1 populations, but not in the mNK2 population. This is consistent with a defect at the mNK1 to mNK2 transition that has previously been reported in the absence of Tbet.^18^

The accumulation of NK cells in the bone marrow of both strains of Tbet‐deficient mice, together with the underexpression of *S1pr5* mRNA reported in Duane NK cells and the defect in bone marrow egress displayed by *S1pr5* ^−/−^ NK cells, led to the suggestion that Tbet mediates NK cell bone marrow egress via S1PR5.^12^, ^19^, ^20^ However, no previous study had formally addressed the question of whether the accumulation of NK cells in the absence of Tbet occurs as the result of a migration defect. *In vivo* staining of sinusoidal cells is widely accepted as an indicator of bone marrow egress and we used this approach to show that *Tbx21* ^−/−^ NK cells are less able to move from the parenchyma to the sinusoid of the bone marrow, supporting the idea that Tbet promotes NK cell bone marrow egress.

A possible confounding factor in this analysis is the extent to which the block at the mNK1 to mNK2 transition could account for the accumulation of mNK1 cells in the parenchyma of these mice, rather than a defect in migration. Although the block in development may contribute to the accumulation in the parenchyma, we believe it is unlikely to be the key determinant of the phenotype in the parenchyma because, were this the case, we would also expect to see an accumulation of mNK1 in the sinusoid, whereas we actually observed a decrease.

After we confirmed that the NK cell accumulation in *Tbx21* ^−/−^ bone marrow was caused by defective migration, we explored which mediators were involved, identifying a transcriptional signature associated with Tbet expression in developing NK cells. We confirmed previous reports that *S1pr5* is reduced in Tbet‐deficient NK cells at the transcriptional level,^12^ but we were unable to corroborate this at the protein level. *Cx3cr1* transcription differed between *Tbx21* ^−/−^ and wild‐type cells, but we were unable to find a difference in protein expression. CX3CR1‐deficient mice display a slight accumulation of mature KLRG1^+^ NK cells in the bone marrow, suggesting that if CX3CR1 is involved in bone marrow egress, it is likely to be in the most mature NK cells, which are absent in *Tbx21* ^−/−^ mice.^24^ This is consistent with our finding that CX3CR1 was only detectable in mNK2. We also did not observe significant differences in CCR2 protein expression: based on the regulation of *Ccr2* transcript we would expect to see an increase. CCR2 is important for the egress of a number of immune cell lineages from the bone marrow and is sometimes only detectable intracellularly.^25^ Therefore, one possibility is that *Ccr2* is up‐regulated in the absence of Tbet as a compensatory mechanism, but we failed to detect an intracellular increase in CCR2 levels because we only examined cell surface expression of the protein.

On the other hand, we were able to detect differences between wild‐type and *Tbx21* ^−/−^ NK cells in the protein levels of a number of molecules involved in cell migration. We observed increased expression of CD69 and CD62L, which would be expected to retain cells in the parenchyma,^26^ as well as decreased expression of CXCR6.

We have previously shown that CXCR6 is important for ILC progenitors leaving the bone marrow^23^ so we went on to investigate the role of CXCR6 in NK cell bone marrow egress. In the bone marrow of CXCR6‐deficient mice, we found an accumulation of iNK cells, although this was only significant among *Cxcr6*‐gfp‐expressing cells, consistent with our observation that CXCR6 expression was both highest and most differentially expressed in iNK cells. We did not observe an accumulation among the CD11b^+^ mNK populations. Therefore, the NK cell accumulation we observed in the absence of CXCR6 was less pronounced than that in the absence of Tbet and was only observed in early developmental stages. In competitive reconstitution experiments, we were able to detect a decreased ability of CXCR6‐deficient progenitors to reconstitute the peripheral NK cell compartment, compared with wild‐type cells. Taken together, these observations suggest that, similar to ILC progenitors, bone marrow egress of iNK cells may be promoted by CXCR6. It will be interesting to follow this up with more definitive studies into any *in vivo* egress phenotype, similar to those we report here for Tbet. However, the egress phenotype we report in the absence of Tbet affects mNK1 cells more strongly than iNK cells, suggesting that targets of Tbet, which are most differentially expressed at the mNK1 stage, such as *S1pr5*, *Cx3cr1*, *Sell* and *Cd69*, are likely to be more important for the egress phenotype in the *Tbx21* ^−/−^ mouse than *Cxcr6*.

Overall, we have definitively shown that Tbet is required for NK cells to leave the bone marrow and defined a module of genes, co‐ordinated by Tbet, which are likely to mediate the bone marrow egress phenotype. Among these is *Cxcr6*, the absence of which has a minor impact on iNK egress, and this is reflected in a slight defect in the ability of CXCR6‐deficient NK cells to reconstitute the periphery. However, the egress phenotype of the Tbet knockout is more pronounced in mNK1 than iNK cells, suggesting that the targets of Tbet that are differentially expressed at the mNK1 stage are likely to be the major mediators of the phenotype. As we move into an era where the cancer‐fighting potential of NK cells is being harnessed to develop immunotherapies, it will become increasingly important to understand NK cell trafficking in order to maximize the full potential of those therapies.^27^ It will also be useful, in the future, to better define the roles of some of the other genes that we identified as targets of Tbet in NK cell bone marrow egress.

## Author contribution

RG and VM designed the study. AOC, TP and SD carried out experiments. AOC, TP, RG and VM analysed data. AOC and VM wrote the paper. All authors contributed to the manuscript.

## Conflict of interest

The authors declare no conflict of interests.

## Supporting information


**Figure S1**. Tbet expression in natural killer cell developmental intermediates.
**Figure S2**. Selected positive and negative controls for flow cytometry staining.

## Data Availability

Raw RNAseq data and differentially expressed gene lists are available from the National Center for Biotechnology Information Gene Expression Omnibus under accession no. GSE122874; https://www.ncbi.nlm.nih.gov/geo/query/acc.cgi?acc=GSE122874
