# Supplementary material for: Tbet promotes CXCR6 expression in immature natural killer cells and natural killer cell egress from the bone marrow
Source: Immunology. 2020 Jun 8;161(1):28–38. doi: 10.1111/imm.13204 (PMC7450165; doi:10.1111/imm.13204)
Supplement: Supplementary file 1 — Figure S1. Tbet expression in natural killer cell developmental intermediates. Figure S2. Selected positive and negative controls for flow cytometry staining. [file IMM-161-28-s001.pdf]

Supplementary Information

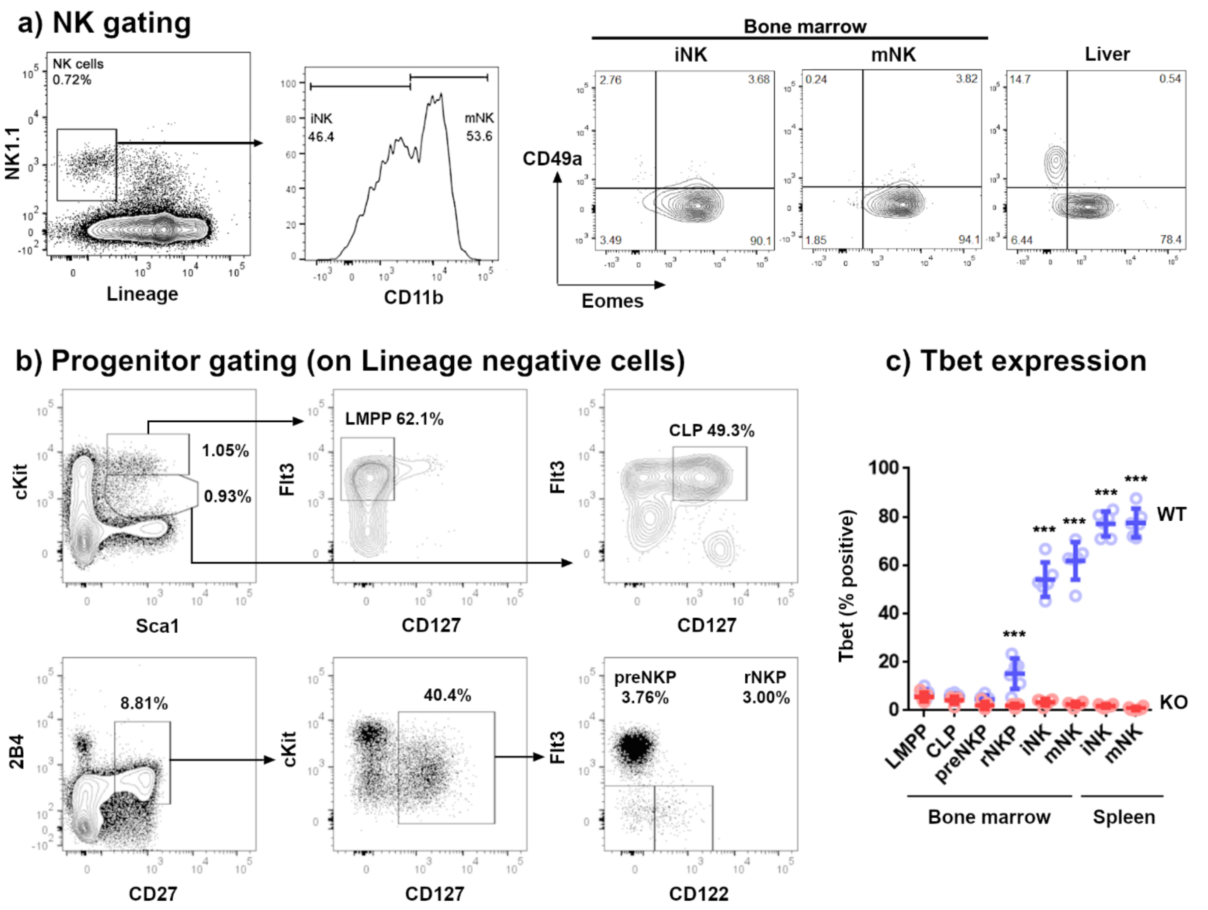

Supplementary Figure 1. Tbet expression in NK cell developmental intermediates

(a) Flow cytometry gating strategy identifying NK cells in the bone marrow. NK cells were identified by gating on single, live, CD45+ cells and by leukocyte scatter, lineage negative and NK1.1+. iNK and mNK were identified by differential CD11b expression. Among subsets identified in this way, no significant CD49a+ Eomes- ILC1 population was present. Total lineage negative NK1.1+ cells in the liver, among which ILC1 are prominent, are shown for comparison. (b) Gating strategy for identification of LMPPs, CLPs and NK progenitors (Fathman et al, 2011). (c) Tbet expression over NK cell development in Tbx21+/+ (WT) compared to Tbx21-/- (KO) mice. n = 6, means and SD are shown. Significance was determined using one-tailed t tests with Holm-Sidak correction. * $p < 0.05$; ** $p < 0.01$; *** $p < 0.001$.

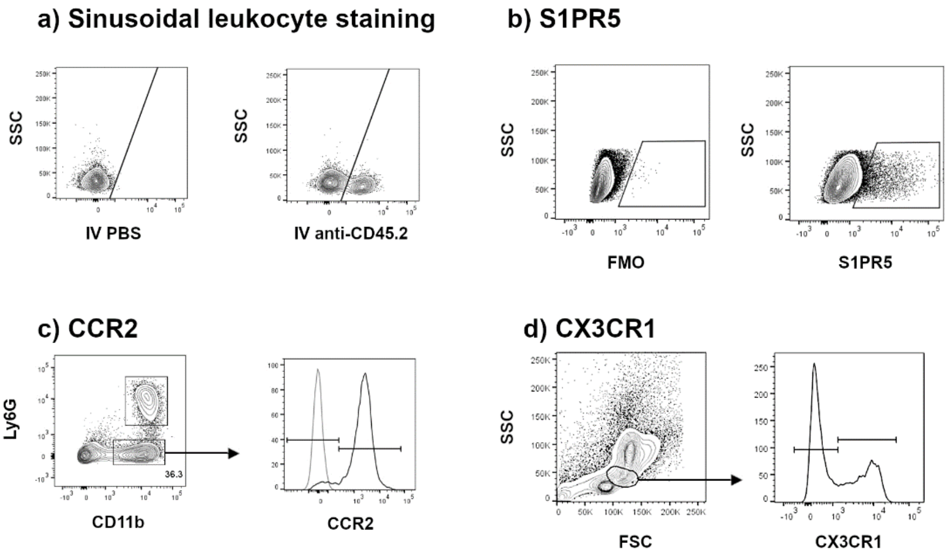

Supplementary Figure 2. Selected positive and negative controls for flow cytometry staining
Positive and negative controls are shown for stains in which clear positive and negative populations are not visible in the figures in the main body of the manuscript. (a) Mice were injected intravenously with PBS (left) or fluorescently-conjugated anti-CD45.2 (right). (b) HEK293T cells were transfected with *S1pr5*. The FMO (left) and antibody-stained cells (right) are shown. (c) CD11b^{+} Ly6G^{-} bone marrow monocytes (left) were stained with anti-CCR2 (solid trace) or FMO (dotted trace). (d) Within the monocyte scatter gate (left) clear negative and positive populations for CX3CR1 (right) are visible.
